# Paternal Care Impacts Oxytocin Expression in California Mouse Offspring and Basal Testosterone in Female, but Not Male Pups

**DOI:** 10.3389/fnbeh.2018.00181

**Published:** 2018-08-29

**Authors:** Christine N. Yohn, Amanda B. Leithead, Julian Ford, Alexander Gill, Elizabeth A. Becker

**Affiliations:** ^1^Department of Psychology, Saint Joseph’s University, Philadelphia, PA, United States; ^2^Department of Psychology, Rutgers University, Piscataway, NJ, United States

**Keywords:** oxytocin, testosterone, corticosterone, paternal care, *Peromyscus californicus*

## Abstract

Natural variations in parenting are associated with differences in expression of several hormones and neuropeptides which may mediate lasting effects on offspring development, like regulation of stress reactivity and social behavior. Using the bi-parental California mouse, we have demonstrated that parenting and aggression are programmed, at least in part, by paternal behavior as adult offspring model the degree of parental behavior received in development and are more territorial following high as compared to low levels of care. Development of these behaviors may be driven by transient increases in testosterone following paternal retrievals and increased adult arginine vasopressin (AVP) immunoreactivity within the bed nucleus of the stria terminalis (BNST) among high-care (HC) offspring. It remains unclear, however, whether other neuropeptides, such as oxytocin (OT), which is sensitive to gonadal steroids, are similarly impacted by father-offspring interactions. To test this question, we manipulated paternal care (high and low care) and examined differences in adult offspring OT-immunoreactive (OT-ir) within social brain areas as well as basal T and corticosterone (Cort) levels. HC offspring had more OT-ir within the paraventricular nucleus (PVN) and supraoptic nucleus (SON) than low-care (LC) offspring. Additionally, T levels were higher among HC than LC females, but no differences were found in males. There were no differences in Cort indicating that our brief father-pup separations likely had no consequences on stress reactivity. Together with our previous work, our data suggest that social behavior may be programmed by paternal care through lasting influences on the neuroendocrine system.

## Introduction

Variability within postnatal environments can have profound consequences on phenotype development in offspring. Stress reactivity and parental behavior, among other things, are programmed by the quality of care received (Bester-Meredith and Marler, 2003; Shannon et al., 2005; Ichise et al., 2006; Uriarte et al., 2007; Rosenfeld et al., 2013). This phenotypic plasticity is accompanied by changes to neural pathways associated with behavioral regulation (Champagne et al., 2004; Weaver et al., 2006; Oomen et al., 2009). While mothers are the primary caregiver in most mammalian species, in an estimated 5%–10% of mammals, fathers also contribute to offspring development (Gubernick and Alberts, 1987; Ziegler et al., 2000; Bester-Meredith and Marler, 2001). Within the bi-parental California mouse (*Peromyscus californicus*), fathers provide high-care (HC) towards both same and opposite sex offspring, which influence neuroendocrine mechanisms that facilitate similar rather than sexually dimorphic development. Transient increases in testosterone levels (Becker et al., 2010; Chary et al., 2015) and greater arginine vasopressin (AVP) expression in the bed nucleus of the stria terminalis (BNST; Frazier et al., 2006; Yohn et al., 2017), accompany territorial aggression in HC offspring. Whereas in most species parental behavior is accompanied by a reduction in aggression and T, in bi-parental species (Hume and Wynne-Edwards, 2005), like the territorial California mouse (Trainor and Marler, 2001, 2002), T remains high in fathers and is important for maintaining paternal behavior since castration reduces paternal behavior in this species (Trainor and Marler, 2001). The current study aimed to identify whether paternal care, which we have demonstrated programs both territoriality (Yohn et al., 2017) and parental behavior (Bester-Meredith and Marler, 2003; Gleason and Marler, 2013; Becker, unpublished; Leithead, unpublished) in adult California mouse offspring, influences other neuroendocrine mechanisms in addition to T and AVP that may act or interact to shape adult behavior.

The neuropeptide oxytocin (OT) is a likely candidate since it is synthesized in the paraventricular nucleus (PVN) and supraoptic nucleus (SON), with projections to social brain areas (Champagne et al., 2001 *rats*; Pedersen and Boccia, 2003 *rats*) that regulate social behaviors (Consiglio et al., 2005 *rats*) including parenting (Bales et al., 2007 *prairie voles*; Neumann and van den Burg, 2013 *rats*) and aggression, particularly parental aggression (Bosch, 2013 “*rodents*”) and hypothalamus-pituitary-adrenal (HPA) function (Neumann et al., 2000 *rats*; Engelmann et al., 2006 *rats*; Rault et al., 2013 *pigs*). Moreover, the OT system is sensitive to gonadal steroids, such as T (reviews see Pedersen, 1997; Sladek et al., 2000 *rat*; Gordon et al., 2011), which alone or by aromatization into estradiol acts as a modulator of OT secretion and receptor expression within brain areas (i.e., hypothalamus) that regulate both reproductive and parenting behavior (Johnson et al., 1991 *rats*; Okabe et al., 2013 *mice*; Gordon et al., 2017 *humans*). Furthermore, there is significant overlap in expression of OT and aromatase enzymes within the mammalian brain, with aromatase also mainly expressed within the hypothalamus and limbic system (Naftolin et al., 2001; Trainor et al., 2006; El-Emam Dief et al., 2013). Furthermore, developmental studies, in mandarin voles, indicate paternally deprived offspring have lower OT receptor expression than offspring raised with a father (Wang et al., 2012; Cao et al., 2014). Whether this is due to the absence of the caregiver or a particular behavior displayed by the father is unknown.

In addition to changes in the brain, environmental influences on social behaviors and stress reactivity may be mediated by alterations to endocrine systems (Bale, 2006; Clinton et al., 2008; Lajud et al., 2012; Carini and Nephew, 2013). For example, California mouse offspring experience transient increases in T in response to paternal retrievals (Becker et al., 2010; Chary et al., 2015). It is possible that experiencing these brief increases in T will result in overall increased basal T levels in adulthood since postnatal T is correlated with adult T (Sachser and Proöve, 1988; Lürzel et al., 2010), although this hypothesis has yet to be tested. Additionally, paternal deprivation leads to deficits in social behavior (Yu et al., 2012; Bambico et al., 2015) and increased anxiety (McEwen, 2007; Roberts et al., 2007; Jia et al., 2009; Kim et al., 2013), which may correlate with HPA dysregulation since in response to stress, corticosterone (Cort) is secreted. However, no transient differences in Cort in response to paternal care (Becker et al., 2010; Chary et al., 2015) nor basal Cort dissimilarities between paternal absence or presence (Wang et al., 2012) have been reported.

Postnatal paternal care impacts the development of social behaviors and may be regulated by a complex interplay between the hormones OT, T and Cort. For instance, OT expression can be increased via T (El-Emam Dief et al., 2013), and then have a buffering effect on the HPA axis, leading to a decrease in Cort release (Leuner et al., 2012). To examine long-term effects of postnatal paternal interactions on neuroendocrine system development, we manipulated California mouse offspring rearing conditions to receive either HC or low-care (LC). Given that removal of the father leads to decreased OT expression (Wang et al., 2012; Cao et al., 2014), we aimed to assess whether variability in paternal care leads to plasticity within the OT system. In the current study, our primary aim was to assess paternal care impact on OT-immunoreactive (OT-ir) cell distribution in the PVN and SON, with these three areas of the brain sensitive to gonadal steroids (El-Emam Dief et al., 2013) and important in regulating stress response (Dabrowska et al., 2011). Therefore, we also assessed adult basal T and Cort levels since the paternal care manipulation can also have long-term effects on the endocrine system. We hypothesized that PVN and SON OT-ir would be higher in HC than LC offspring. Since California mouse pups experience transient increases in T following paternal retrievals (Becker et al., 2010; Chary et al., 2015) and postnatal surges in T lead to higher adult T levels (Sachser and Proöve, 1988; Lürzel et al., 2010), we predicted higher basal T levels in adult HC than LC offspring. Lastly, as a manipulation check, we measured basal Cort levels to confirm that our modified retrieval manipulation had no lasting effects on stress reactivity; predicting similar basal Cort levels between HC and LC offspring.

## Materials and Methods

This study was carried out in accordance with the recommendations of the National Institutes of Health Guide for the Care and Use of Laboratory Animals. The protocol was approved by the Saint Joseph’s University IACUC. Detailed materials and methods are provided as Supplementary Material Information.

### Subjects

Brains were collected from 52 California mice adults (120 days) from the same cohort of mice used in Yohn et al. (2017) study. Briefly, experimental animals postnatal days (PND) 15–21 were assigned randomly to either HC (*n* = 26) or LC (*n* = 26) postnatal paternal rearing conditions. As previously described by Yohn et al. (2017), we used a modified retrieval manipulation (use of Plexiglas divider) to ensure populations of experimental animals had distinct early life experiences (HC and LC). To establish populations, experimental families were first moved into an observational cage on PND 8. Subsequently, paternal retrieval behavior was manipulated for 20 min daily, across PND 15–21 when pups are more inclined to leave the nest and paternal retrieval behavior is at its highest (Bester-Meredith et al., 1999). During each daily manipulation the mother and non-experimental pups were removed and housed separately. The experimental pup was handled for 30 s and returned to the cage either inside the nest (LC) or outside of the nest (HC). Prior to the pups return to the cage, a Plexiglass divider was inserted into the cage separating the large and small compartments of the observational cage. Results from Yohn et al. (2017) showed that across 7 days, HC experienced greater paternal retrievals than LC offspring since the Plexiglass divider (had plastic mesh barrier for LC group) inhibited father-pup contact during the daily LC manipulations. Separation of father and pup had no effect on paternal behavior in the LC group once the father and pup were reunited (Yohn et al., 2017).

### Immunohistochemistry

Adult experimental mice taken from the colony room were euthanized via rapid decapitation. Brains were extracted, immediately fixed in 5% acrolein overnight at 4°C, transferred to a 20% sucrose buffer solution and refrigerated for 48 h, and finally frozen on dry ice and stored at −80°C until cutting. Beginning from approximately Bregma 0.37 through −1.23 (Paxinos et al., 2007; Campi et al., 2013), brains were sliced on a cryostat at 40 um and stored at −80°C in cyroprotectant until staining. Sections were incubated overnight in a previously validated antibody (Trainor et al., 2010) rabbit anti-OT (1:1,000, AB911, Millipore, Temecula, CA, USA) and then for 2 h in goat anti-rabbit IgG (1:250, PI-1,000, Vector Labs, Burlingame, CA, USA) both times diluted in 2% normal goat serum (NGS) phosphate buffer saline (PBS). Next, sections were amplified in Avidin Biotin Complex (Vector Labs, Burlingame, CA, USA) and then visualized using DAB peroxidase substrate kit (Vector Labs, Burlingame, CA, USA). PBS washes occurred before and after incubation, amplification and visualization.

### Image Analysis

**Figure 1:**
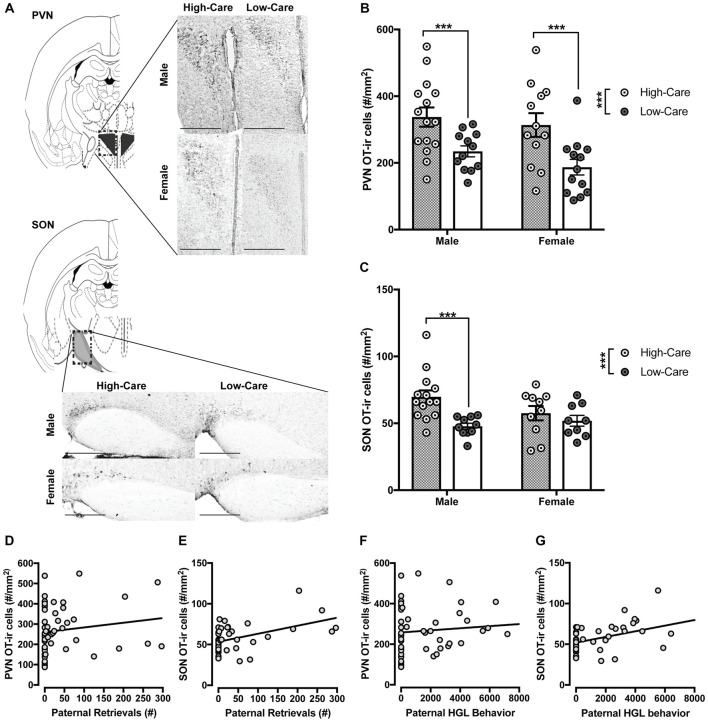
Differences in distribution of oxytocin-immunoreactive (OT-ir) cells between high-care (HC) and low-care (LC) offspring. Representative images of OT-ir staining for each area of interest (atlas images were reproduced from Paxinos mouse atlas, 2007; 10× magnification with scale bar = 500 μm) **(A)**. HC male and female offspring have higher OT-ir cells within the paraventricular nucleus (PVN; **B**) and supraoptic nucleus (SON; **C**) compared to LC offspring. Number of postnatal paternal is significantly associated with amount of OT-ir cells within the PVN **(D)** and SON **(E)**. Amount of postnatal paternal huddling, grooming and licking (HGL) behavior also was significantly associated with distribution of OT-ir cells in these areas **(F,G)**. ***p-value < 0.001, *p-value < 0.05.

Sections were photographed on a Leica DM 2000 outfitted with a DFC310 FX digital color camera (Leica) at 10× magnification. For all cell counts, number of OT-ir positive cells were averaged across two images of the PVN (posterior, Bregma −0.70 thru −94) and SON (Bregma −0.70 thru −94; Figure 1A).

### Testosterone and Corticosterone Enzyme Immunoassay

Trunk blood was collected at brain extraction, with enough serum from 39 of 52 experimental mice (male = 20, female = 19). After collection, samples were centrifuged and separated, then stored at −80°C until assayed. Plasma T (1:10 dilution) and Cort (1:50 dilution) concentrations were determined using commercial assay kits (Enzo Life Sciences, Farmingdale, NY, USA) previously validated in the California mouse (Chary et al., 2015). The standard curve slope generated for Cort had a slope of 1 (*r*^2^ = 0.91) and the slope for T was 0.72 (*r*^2^ = 0.87). Inter-assay coefficient of variability values were 1.1% (Cort) and 3.1% (T) with intra-assay coefficient of variability values being 1.87% (Cort) and 2.65% (T). The cross-reactivity of the Cort kits, according to the manufacturer, was 100% for Cort, 28.6% for deoxycorticosterone, 1.7% for progesterone, and negligible for other steroid hormones (>1%). The cross-reactivity of the T kits was 100% for T, 14.64% for 19-hydroxytestosterone, 7.20% for androstendione, and negligible for other steroid hormones (>1%). Kit sensitivity was 26.99 pg/mL for Cort and 5.67 pg/mL for T.

### Statistical Analyses

Separate 2 × 2 analysis of variance (ANOVA) were run to assess early life rearing conditions and sex differences on PVN and SON OT-ir and basal T and CORT levels. *Post hoc* independent samples *t*-tests for within-sex differences were run. Pearson’s correlations were run to assess relationship between paternal behavior and expression of OT-ir within each area. One animal (female LC) was removed from analyses as cell counts were only obtained from one of the three regions and T levels were three standard deviations above the mean. All statistical analyses were conducted using SPSS (version 23.0, Chicago, IL, USA).

## Results

### OT-Immunoreactivity

Separate ANOVAs indicated HC offspring had significantly more OT-ir in the PVN (*F*_(1,48)_ = 17.49, *p* < 0.001; Figure 1B) and SON (*F*_(1,39)_ = 9.1, *p* = 0.004; Figure 1C) than LC offspring. Planned *post hoc* comparisons revealed HC males had significantly more OT-ir than LC males within the PVN (*t*_(25)_ = 2.89, *p* = 0.008; Figure 1B) and SON (*t*_(22)_ = 3.6, *p* = 0.002; Figure 1C). Within females, HC offspring had more OT-ir in the PVN (*t*_(23)_ = 3.01, *p* = 0.006; Figure 1B) than LC offspring. Unlike males, SON OT was similarly expressed between HC and LC females (*p* = 0.421; Figure 1C). We observed no effect of sex nor an interaction between rearing condition and sex on OT-ir within these regions (*p*’s > 0.2).

### Relationship Between OT-Immunoreactivity and Early Life Rearing Conditions

Paternal retrievals were positively correlated with PVN (*r* = 0.44, *p* = 0.02; Figure 1D) and SON (*r* = 0.47, *p* < 0.001; Figure 1E) OT-ir. Additionally, paternal huddling, grooming and licking (HGL) behavior was positively correlated with PVN (*r* = 0.34, *p* = 0.016; Figure 1F), SON (*r* = 0.44, *p* = 0.003; Figure 1G) OT-ir.

### Plasma Corticosterone and Testosterone Concentrations

**Figure 2:**
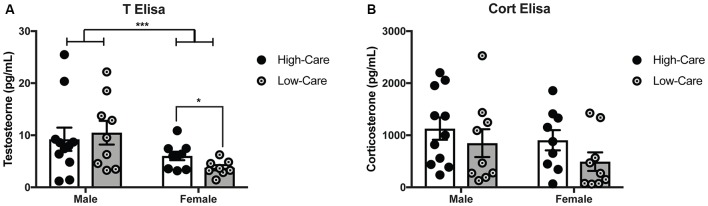
Differences in early life paternal care on hormonal levels. HC females have higher T levels than LC females, with males having higher overall testosterone levels females **(A)**. Corticosterone (Cort) levels did not differ between HC and LC adult offspring across both sexes **(B)**. ***p-value < 0.001, *p-value < 0.05.

As expected, males had higher plasma T levels than females (*F*_(1,33)_ = 7.58, *p* = 0.009; Figure 2A). While there was no main effect of rearing condition on plasma T concentrations (*p* = 0.7) nor differences among males (*p* = 0.69); rearing effects were indicated with higher T levels in HC than LC females, (*t*_(15)_ = 2.27, *p* = 0.039). No rearing effects nor an interaction effect on plasma T levels (*p*’s > 0.05) were found.

No rearing effects, sex differences, nor an interaction were found for plasma Cort concentrations (*p*’s > 0.05; Figure 2B).

## Discussion

Variability in paternal care produced differences in offspring OT-ir. Pups raised under HC conditions had greater OT-ir than LC offspring within the PVN and SON, regions that regulate various behaviors ranging from parenting (Neumann and van den Burg, 2013; Wang et al., 2015) to affective behaviors and autonomic functions (Cao et al., 2014; Yee et al., 2016). Within the PVN, OT expression is linked to the onset and maintenance of both maternal (Neumann and van den Burg, 2013) and paternal care (Kenkel et al., 2014). Since paternal care programs adult offspring social behavior in the California mouse (Bester-Meredith and Marler, 2001; Frazier et al., 2006; Yohn et al., 2017), we suggest that these differences in OT may guide the development of these distinct behavioral phenotypes. Previous studies delineate the relationship between paternal care and OT receptor expression within the PVN and SON (Wang et al., 2012; Cao et al., 2014), our novel findings illustrate susceptibility of PVN, and SON OT-ir to postnatal paternal care. However, unlike paternal deprivation studies, we emphasize the importance of paternal behaviors on adult OT-ir with high levels of both paternal retrievals and HGL behavior positively related to PVN and SON OT-ir. In response to paternal retrievals HC offspring experience transient increases in T (Becker et al., 2010; Chary et al., 2015), with OT expression within these brain areas regulated in part by T (Sladek et al., 2000; Gordon et al., 2011) and the aromatization of T into estradiol (Naftolin et al., 2001; Trainor et al., 2006; El-Emam Dief et al., 2013). The PVN and SON contain high levels of aromatase (El-Emam Dief et al., 2013), which could further explain differences in PVN and SON OT-ir between HC and LC offspring. In the California mouse male and female offspring retrieve their offspring at similar levels as they received during development (Bester-Meredith and Marler, 2003; Becker, unpublished; Leithead, unpublished), however mechanisms for this behavioral transmission are not fully elucidated. Since OT receptor expression is related to level of postnatal care (Francis et al., 2002; Perkeybile et al., 2015), our results suggest a potential mechanism by which parental care is transmitted across generations.

Since sex differences in OT are reported (Lee et al., 2009; Carter, 2014), we tested for paternal effects on OT-ir within each sex even though no overall sex differences were observed. Our analyses revealed HC males and females had more OT-ir within the PVN than LC offspring, suggesting OT-ir is plastic in response to the environment, potentially allowing more adapted social behaviors within HC offspring. However, within the SON, only males were susceptible to variability in care, which may be due to sex differences in social behaviors and physiological functions that SON OT-ir regulates. In females and males SON OT is associated with parenting and other social behaviors (Song et al., 2010; Bales et al., 2011); with SON OT also facilitating uterine contraction and lactation in females (Higashida et al., 2013). While future maternal care may be susceptible to postnatal rearing conditions, other functions like uterine contractions and lactation may be resistant to environmental fluctuations in OT, thus resulting in HC and LC females having similar expression within the SON. Alternatively, this null result could have been confounded by estrous, since SON OT-ir fluctuates in relation to circulating estrogen levels (Shughrue et al., 2002) and we did not track estrous cycle.

Since paternal retrievals induce transient increases in T (Becker et al., 2010; Chary et al., 2015) and postnatal surges in T, may be related to adult T levels (Sachser and Proöve, 1988; Lürzel et al., 2010), we wanted to assess long-term effects of rearing condition on adult basal T levels. Not surprisingly, we found males had higher basal T levels than females. Within females, we observed greater T levels in HC than LC offspring, which is likely a long-term effect of rearing condition. Consistent with Wang et al. (2015) prairie vole study, we found no differences in male T. It is possible that postnatal paternal interactions may not have long term effects on male T, or since males already have high T, that a ceiling effect (Evans et al., 2000) may obscure a potential impact. Seeing as OT expression is associated with a buffering effect on HPA function (Neumann et al., 2000; Leuner et al., 2012) and T can have organizational effects on the HPA axis (Seale et al., 2005; Goel and Bale, 2008), we examined adult basal Cort levels as a manipulation check since parental interactions may influence HPA function (Slotten et al., 2006; Engert et al., 2011). Consistent with paternal deprivation studies and our previous work (Becker et al., 2010; Wang et al., 2012; Chary et al., 2015; Yohn et al., 2017) basal Cort levels were similar between HC and LC offspring, suggesting our manipulation had no lasting effects on Cort. To further test the impact of our manipulation on stress responsivity, future studies could measure Cort levels in adult mice after experiencing a stressful situation.

Our results demonstrate developmental plasticity within the OT system in response to the postnatal paternal environment which may be mediated by transient changes in T subsequent to paternal retrievals during development. Our study is the first to illustrate long-term effects of paternal care on basal T levels in females, which may mediate transmission of social behaviors, like parenting and aggression in territorial species. Future studies are needed to examine the relationship between postnatal increases in T in response to paternal retrievals and adult OT expression to delineate whether the interplay between postnatal T and adult OT mediate changes in social behavior. Our results emphasize the critical role fathers hold in the development of the neuroendocrine system in both males and females.

## Author Contributions

CY designed, conducted the behavioral manipulations, collected both the tissue and plasma used in this study. Additionally, CY ran all the statistical analyses and wrote the manuscript. AL assisted in behavioral manipulations, tissue collection and in conducting the Elisa’s. She also helped in the manuscript preparation by giving thoughtful feedback/input on each draft. JF contributed to this project through conducting the immunohistochemistry and imaging all stained sections. AG assisted with this project by scoring behavioral videos collected during each of the 7 days of experimental manipulation. He also assisted with the Elisa’s. EB was the principal investigator of this project and gave guidance/assistance on all phases of this project. Additionally, she assisted in the manuscript preparation.

## Conflict of Interest Statement

The authors declare that the research was conducted in the absence of any commercial or financial relationships that could be construed as a potential conflict of interest.

## Abbreviations

AVP: arginine vasopressin
BNST: bed nucleus of the stria terminalis
CORT: corticosterone
HC: highcare
HGL: huddling, licking and grooming behavior
HPA: hypothalamic-pituitary-adrenal
-ir: immunoreactive
LC: lowcare
NGS: normal goat serum
OT: oxytocin
PBS: phosphate buffer saline
PVN: paraventricular nucleus
SON: supraoptic nucleus
T: testosterone.
