# Supplementary material for: Paternal Care Impacts Oxytocin Expression in California Mouse Offspring and Basal Testosterone in Female, but Not Male Pups
Source: Front Behav Neurosci. 2018 Aug 29;12:181. doi: 10.3389/fnbeh.2018.00181 (PMC6123359; doi:10.3389/fnbeh.2018.00181)
Supplement: Supplementary file 1 [file Data_Sheet_1.PDF]

Supplementary Material

Brief Research Report

Christine N. Yohn*[Ψ§], Amanda B. Leithead[Ψ], Julian Ford [Ψ] Alexander Gill [Ψ], Elizabeth A. Becker[Ψ]

Correspondence: Christine N. Yohn: cy253@scarletmail.rutgers.edu

Methods

Subjects

For the current study, brains were collected from 52 adult (120 days of age) California mice (*P. californicus*) used in a previous study (Yohn et al., 2017). Briefly, animals were assigned randomly to either high paternal care (HC, n=27) or low paternal care (LC, n=25) rearing conditions. Across 7 days of manipulations, HC offspring experienced greater paternal retrievals than LC offspring. For detailed methods see Yohn and colleagues (2017). At weaning, animals were housed in same-sex groups of 2-3 in standard opaque cages (48.3 cm × 26.7 cm × 15.6 cm) with aspen shavings, a nestlet (Ancare Corp.,) and ad libitum access to food (Purina 5001 mouse chow) and water. Research animals were maintained under a 14 L: 10D reverse light dark cycle, with sustained ambient temperatures between 18 and 22 degrees Celsius and treated in accordance with National Institute of Health Guide for the Care and Use of Laboratory Animals. All procedures were approved by the Saint Joseph's University Institutional Animal Care and Use Committee.

Immunohistochemistry

The impact of early life rearing condition on oxytocin immunoreactivity was assessed with immunohistochemistry. At 120 days of age, experimental males (n=26) and females (n=26) were euthanized via rapid decapitation. Brains were collected and immediately fixed in 5%

acrolein overnight at 4°C. On the following day, brains were transferred to a 20% sucrose buffer solution and refrigerated for 48 h. Brains were then frozen on dry ice, wrapped in parafilm and stored at −80 °C until cutting. Beginning from approximately Bregma 0.37 through −1.23 (Paxinos mouse atlas; Campi et al., 2013), brains were sliced on a cryostat at 40 um and stored at −80 °C in cyroprotectant until staining. Immunostaining for oxytocin (OT) was run on every other section in batches containing at least twelve animals per treatment group (HC and LC), with equal representation of males and females in each group. At room temperature, free-floating tissue sections were washed six times for 5 minutes per wash in phosphate buffer saline (PBS). Next, sections were incubated overnight at 4°C in a primary rabbit anti OT (1:1000, AB911, Millipore, Temecula, CA) previously validated by Trainor and colleagues (2010), diluted in 2% normal goat serum NGS in 0.5% Triton X (PBS-Tx). After 24 hours, tissue was washed six times for 5 min in PBS and then incubated for 2 h at room temperature in goat anti rabbit IgG (1:250, PI-1000, Vector Labs, Burlingame, CA) mixed with 2% NGS PBS. Following three 5-minute PBS washes, sections were amplified for 1 hour in Avidin Biotin Complex (Vector Labs, Burlingame, CA) prepared in PBS. Sections were then subjected to three 5-minute washes in PBS before the antibody complex was visualized using DAB peroxidase substrate kit (Vector Labs, Burlingame, CA) as the chromogen. Finally, sections were washed three times in PBS for 5 min before being mounted onto slides (Fisher, Pittsburgh, PA) and coverslipped using Permount Mounting Medium (Fisher, Pittsburgh, PA).

Image Analysis

Sections were photographed on a Leica DM 2000 outfitted with a DFC310 FX digital color camera (Leica) at 10x magnification. For all cell counts, two images of the PVN, SON and BNST were identified and matched for analysis. Magnocellular and parvocellular neurons were

not isolated within the PVN since there is overlapping in the cellular distribution in *P. californicus* (Steinman et al., 2015; de Jong et al., 2009). A single observer blind to treatment counted the number of OT-immunoreactive (ir) cells within each brain area in a box (0.26 × 0.31 mm) using Image J (NIH, Bethesda, MD; Yohn et al., 2017). To identify positively stained cells, a constant threshold of staining was set across sections and counted using the "analyze particle" function in Image J (Yohn et al., 2017; Trainor et al., 2010). The number of cells was averaged across the 2 sections per brain region, where the average number of cells were divided by mm^2 for each animal before statistical analyses. To ensure reliability, all images counted via threshold were compared to the cell counts of those images conducted by eye (r = 0.76). Furthermore, OT-ir cell sizes were assessed between the groups to ensure cell size did not significantly influence OT-ir cell counts in each area. No differences in OT-ir cell sizes were observed between males and females ($F(1, 43) = 0.57$, $p = 0.45$) and treatment groups ($F(1, 43) = 1.24$, $p = 0.27$).

Testosterone and Corticosterone Enzyme Immunoassay

Trunk blood was collected at the time of brain harvesting. We had enough serum from 39 of the 52 mice used in the experiment (male = 20, female = 19) for analysis. After blood collection, samples were centrifuged and separated. Plasma samples were stored at −80 °C until assayed.

Plasma concentrations of T and Cort were determined using commercial assay kits (Enzo Life Sciences, Farmingdale, NY) previously validated in *P. californicus* (Chary et al.., 2015). For the assays, 50 μL of each plasma sample were used. Hormones were extracted by adding 1 mL of diethyl ether and then thoroughly mixing the solution (Fisher Analog Vortex Mixer). After tubes settled, samples were placed into a bath of 95% ethanol with dry ice and the resulting aqueous solutions were decanted into separate test tubes. Next, the ether evaporated by placing the tubes

into a water bath inside a fume hood. This procedure was conducted twice for each sample to maximize the amount of hormone used for the assay.

Samples were reconstituted with assay buffer provided by the assay kit manufacturer using a 1:10 dilution for T and a 1:50 dilution for Cort. We prepared samples, controls, and standards in duplicate and added in 100μL aliquots to the designated plate wells. Manufacturer instructions were followed for all procedures. Washes were performed using Biotek ELx50Microplate Strip Washer. Sample readings were completed using Biotek ELx808 Absorbance Reader and Gen5 Software (Biotek, Winooski, VT, Version 2.09). Readings for both T and Cort were assessed at 405 nm with a correction between 570 and 590 nm. We assessed percentage-binding data of each sample from the standard curve by plotting against logarithmic transformations of their dosages and the resulting regression equation was compared to those of the dilution sequences. The standard curve slope generated for Cort had a slope of 1 ($r^2 = 0.91$) and the slope for T was 0.72 ($r^2 = 0.87$).

The cross-reactivity of the Cort kits, according to the manufacturer, was 100% for Cort, 28.6% for deoxycorticosterone, 1.7% for progesterone, and negligible for other steroid hormones (>1%). The cross-reactivity of the T kits was 100% for T, 14.64% for 19-hydroxytestosterone, 7.20% for androstendione, and negligible for other steroid hormones (>1%). Kit sensitivity was 26.99 pg/mL for Cort and 5.67 pg/mL for T.
